# Biological and Inflammatory Effects of Antigen 5 from *Polybia paulista* (Hymenoptera, Vespidae) Venom in Mouse Intraperitoneal Macrophages

**DOI:** 10.3390/toxins13120850

**Published:** 2021-11-29

**Authors:** Murilo Luiz Bazon, Luis Gustavo Romani Fernandes, Isabela Oliveira Sandrini Assugeni, Lucas Machado Pinto, Patrícia Ucelli Simioni, Ricardo de Lima Zollner, Márcia Regina Brochetto Braga

**Affiliations:** 1 Laboratório de Biologia Molecular de Artrópodes—LBMA, Instituto de Biologia—IB, Universidade Estadual Paulista Câmpus Rio Claro—UNESP-RC, Avenida 24-A, n° 1515, Bela Vista, Rio Claro CEP 13506-900, SP, Brazil; bazonmurilo@gmail.com (M.L.B.); bela.assugeni@gmail.com (I.O.S.A.); l_machado@hotmail.com (L.M.P.); 2 Laboratório de Imunologia Translacional—LIT, Departamento de Clínica Médica, Faculdade de Ciências Médicas—FCM, Universidade Estadual de Campinas—UNICAMP, Rua Tessália Vieira de Camargo, n° 126, Cidade Universitária “Zeferino Vaz”, Campinas CEP 13083-887, SP, Brazil; luisgrf@unicamp.br (L.G.R.F.); zollner@unicamp.br (R.d.L.Z.); 3 Departamento de Biomedicina, Faculdade de Americana—FAM, Avenida Joaquim Bôer, n° 733, Jardim Luciane, Americana CEP 13477-360, SP, Brazil; psimioni@gmail.com; 4 Faculdade de Medicina, Universidade Anhembi Morumbi, Av. Rio das Pedras, 1601-Pompéia, Piracicaba CEP 13425-380, SP, Brazil

**Keywords:** Hymenoptera, *Polybia paulista*, antigen 5, macrophages, inflammatory effects

## Abstract

The social wasp *Polybia paulista* (Hymenoptera, Vespidae) is highly aggressive, being responsible for many medical occurrences. One of the most allergenic components of this venom is Antigen 5 (Poly p 5). The possible modulation of the in vitro immune response induced by antigen 5 from *P. paulista* venom, expressed recombinantly (rPoly p 5), on BALB/c mice peritoneal macrophages, activated or not with LPS, was assessed. Here, we analyzed cell viability changes, expression of the phosphorylated form of p65 NF-κB subunit, nitric oxide (NO), proinflammatory cytokines production, and co-stimulatory molecules (CD80, CD86). The results suggest that rPoly p 5 does not affect NO production nor the expression of co-stimulatory molecules in mouse peritoneal macrophages. On the other hand, rPoly p 5 induced an increase in IL-1β production in non-activated macrophages and a reduction in the production of TNF-α and MCP-1 cytokines in activated macrophages. rPoly p 5 decreased the in vitro production of the phosphorylated p65 NF-κB subunit in non-activated macrophages. These findings suggest an essential role of this allergen in the polarization of functional M2 macrophage phenotypes, when analyzed in previously activated macrophages. Further investigations, mainly in in vivo studies, should be conducted to elucidate *Polybia paulista* Ag5 biological role in the macrophage functional profile modulation.

## 1. Introduction

Brazil holds a great diversity of clinically significant Neotropical Hymenoptera, including the three prominent families: Vespidae, Formicidae, and Apidae. *Polybia paulista* (Hymenoptera: Vespidae), a Brazilian Neotropical wasp, is responsible for many sting accidents, including fatal anaphylaxis cases [1]. Phospholipase A1 (Poly p 1), hyaluronidase (Poly p 2), and antigen 5 (Poly p 5) are the main allergens present in the *P. paulista* venom. With proteomic approaches, these allergens were characterized [2,3,4]. Ag5 allergens are the most abundant proteins in most Vespoidea venoms, including *P. paulista*, but their function remains unclear [5].

Antigen 5, cysteine-rich secretory proteins (CRISP), and pathogenesis-related proteins (Pr-1) belong to the CAP superfamily and engage in several biological processes, for instance reproduction, malignant tumors, and hypersensitivity reactions [5]. Antigen 5 has been successfully used in molecular diagnosis to differentiate true double sensitization to wasp and honeybee venoms as well as cross-reactivity [6]. As reported in other wasps, Poly p 5 (~23 kDa) is a glycosylated allergen present in large amounts in crude venom and has seven described isoforms [1,4].

Systemic reactions to venom due to Hymenoptera stinging affect the respiratory and vascular system, leading to multiorgan failure [7]. In these reactions, the venom’s allergens trigger responses of the immune system to the generation of specific antibodies, degranulation of mast cells, and the involvement of cells (such as macrophages and lymphocytes) [8]. 

Antigen-presenting cells, such as macrophages, crucially participate in the inflammatory process, initiating the immune response in resistance to allergens by releasing cytokines and presenting the allergens to naive CD4^+^ T cells [9]. A cell signaling cascade is triggered by the attachment of each cytokine to its determined cell surface receptor, causing cell functions to promote the regulation of diverse specific genes related to the modulation of inflammatory responses and their transcription factors. They are indispensable to macrophages activity by giving assistance to the generation of an successful immune response, as well as to the innate and adaptive immunity association, affecting the macrophages’ microenvironment [10].

The study and characterization of different proteins components of insect venom becomes significant to improve diagnostic methods, providing a more accurate understanding of both specificity patterns and cross-reactivity between different allergens [11]. Furthermore, the knowledge of the role of each Hymenoptera venom allergen in the modulation of immune cells is an approach that has been hardly explored in the literature.

Here, we aimed to assess the immunomodulatory properties of Ag5 on the macrophage activation markers products, such as the cytokine secreting profile, co-stimulatory molecules expression, nitric oxide production, and the activation status of the nuclear factor kappa B (NF-κB) transcription factor. The recombinant form of Ag5 (rPoly p 5) was used as a highly purified source of this protein, corresponding to the Poly p 5 allergen of the *Polybia paulista* venom [1]. rPoly p 5 was able to modulate the production of IL-1β and the expression of the phosphorylated form p65 of NF-κB in non-activated macrophages and, on the other hand, modulate the production of TNF-α and MCP-1 cytokines in LPS-activated intraperitoneal macrophages. However, it does not seem to affect NO production or modulate the expression of CD80 and CD86 co-stimulatory molecules.

## 2. Results

### 2.1. Expression and Purification of rPoly p 5

**Figure 1:**
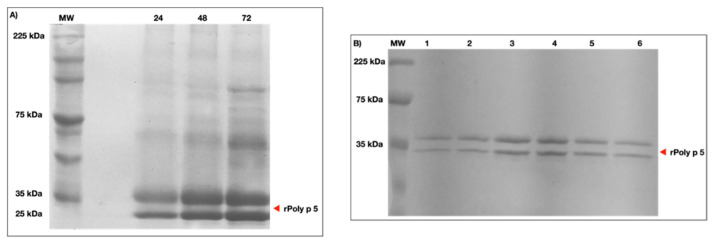
(**A**) Supernatant from *P. pastoris* X-33 culture in the course of rPoly p 5 expression protein profile on SDS-PAGE (12%). Abbreviations: 24–72 h after induction; (**B**). Purification of the recombinant allergen rPoly p 5 by affinity chromatography on Ni-NTA agarose and analysis by SDS-PAGE 12%. (M): Broad-range protein molecular weight markers (A): 1-6: rPoly p 5 samples eluted from Ni-NTA column with 75 mM Imidazole.

To obtain soluble rPoly p5, the supernatant of the *P. pastoris* X-33 culture was evaluated. After 24 h of induction with 1% methanol, SDS-PAGE electrophoresis revealed a band of ~23 kDa, which represents the predicted molecular weight of the protein (Figure 1A). A high concentration of the protein expression was acquired at 72 h. Under ideal circumstances (28 °C, 1% methanol, 250 rpm), rPoly p 5 expression yielded a peak value of 316 mg/L of crude supernatant. Intriguingly, throughout the expression, a distinct band of ~33 kDa with equimolar concentration was also identified. Following rPoly p 5 production, the supernatant was filtered and applied to a commercial prepacked affinity column for chromatography utilizing the N-terminal 6xHis tag provided by the pPICZαA. rPoly p 5 eluted at a concentration of 75 mM of imidazole, according to the results from the collected fractions analysis. After elution, two bands were produced, one with ~23 kDa, representing the expected molecular weight, and the other with ~33 kDa, indicating a variant of the heterologous protein (Figure 1B). This variant occurrence might be related to rPoly p 5 being hyper-mannosylated [12]. The molecular basis for this second band will be discussed ahead in this work. 

### 2.2. Nitric Oxide Production

**Figure 2:**
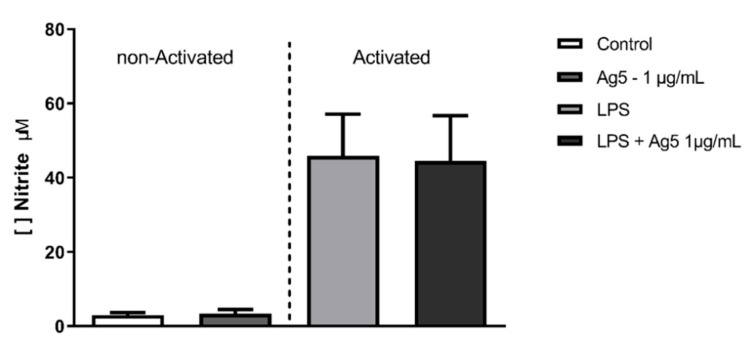
The effect of rPoly p 5 on nitric oxide production by intraperitoneal macrophages stimulated with rPoly p 5. The bars represent the mean ± (S.E.M) of nitrite concentration detected in the cell cultures supernatants of BALB/c mice intraperitoneal macrophages stimulated with the proinflammatory stimulus (LPS at 1 μg/mL—light gray and black bars) or that received no stimulus (white and dark gray bars). Concomitantly with the addition of this stimulus, the cells were incubated with rPoly p 5 at a concentration of 1 μg/mL for 48 h (dark grey and black bars), and the controls received only cell culture medium (white and light grey bars). (*N* = 16).

To the nitric oxide (NO) quantification analysis, a sample of 16 animals was used. LPS-activated and non-activated intraperitoneal macrophages cell cultures, treated with 1 μg/mL of rPoly p 5, were used as the supernatant source to evaluate NO production. As shown in Figure 2, in non-activated macrophages (without LPS stimulation), the dose of 1 μg/mL rPoly p5 does not affect NO production in macrophages when compared with the control group. In contrast, on activated macrophages (LPS treated), we observed a higher nitrite release in culture supernatants than in non-stimulated cultures. Although we verified a higher concentration of NO production in the rPoly p 5 + LPS group than the control without LPS, there was no difference when compared with LPS-stimulated cultures but in privation of rPoly p 5. In summary, these results indicate that rPoly p 5 does not affect NO production in macrophages, neither stimulating the production in non-activated nor downregulating in the LPS activated ones. 

### 2.3. Pro and Antiinflammatory Cytokine Production

**Figure 3:**
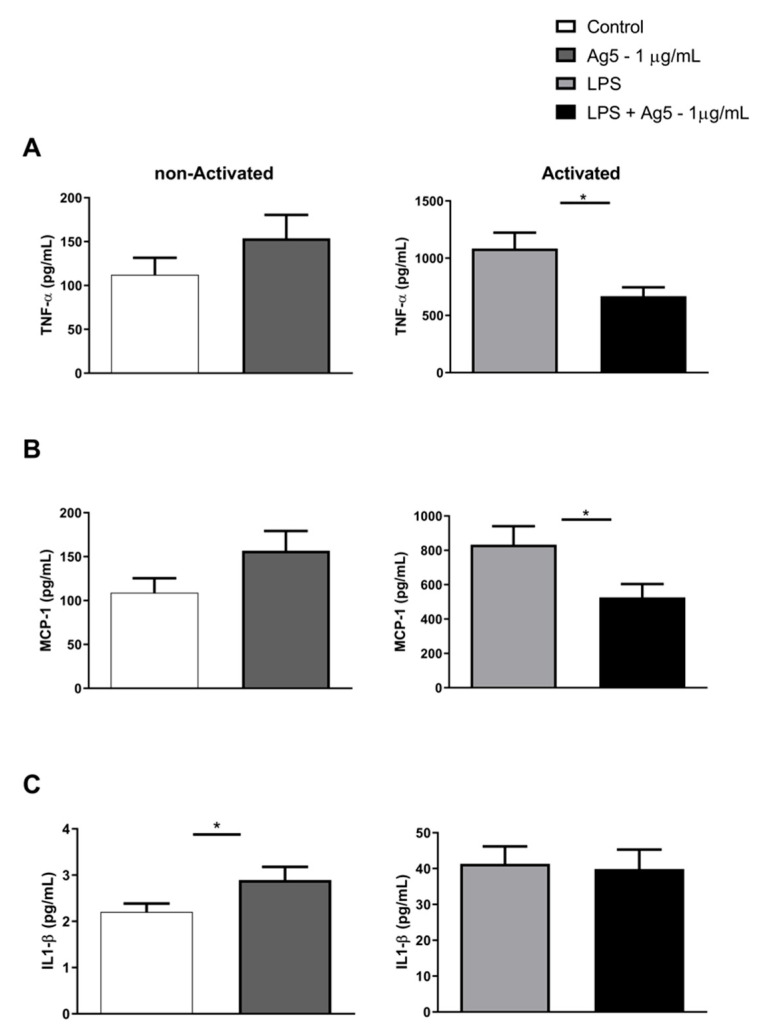
Effect of rPoly p 5 on cytokine production by intraperitoneal macrophages stimulated with rPoly p 5: (**A**) TNF-α, (**B**) MCP1, (**C**) IL1-β. The bars represent the mean ± (S.E.M) of cytokine concentrations detected in the cell cultures supernatants of BALB/c mice intraperitoneal macrophages stimulated with the proinflammatory stimulus (LPS at 1 μg/mL—light gray and black bars), or that without stimulus (white and dark gray bars). Concomitantly with the addition of this stimulus, the cells were incubated with rPoly p 5 at a concentration of 1 μg/mL for 48 h (dark gray and black bars), and the controls received only cell culture medium (white and light grey bars). (*N* = 44) (*) *p* < 0.05—Mann–Whitney U test.

**Figure 4:**
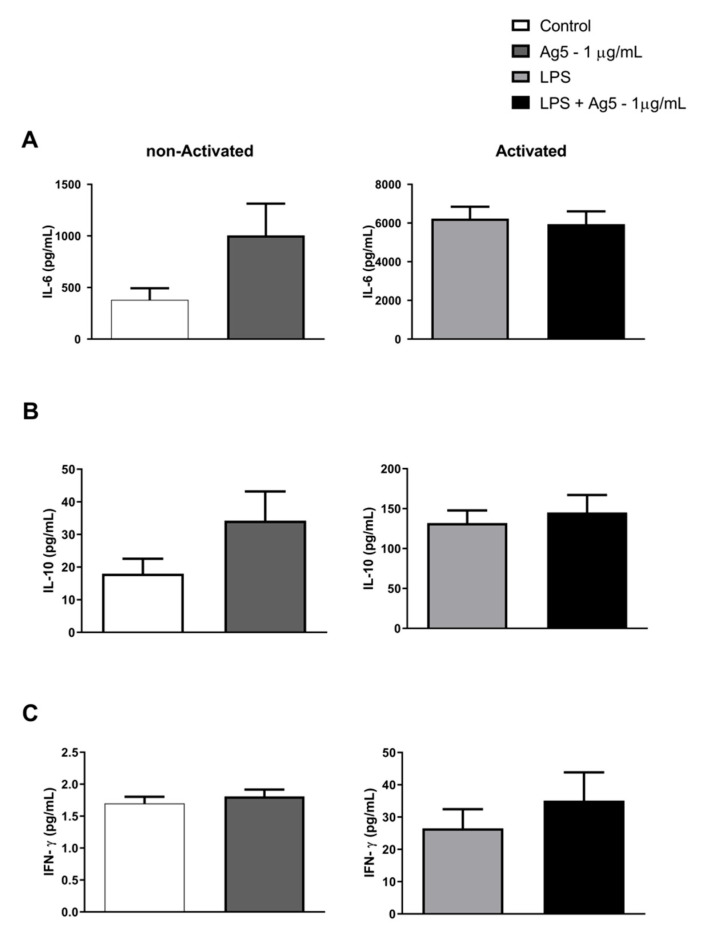
Effect of rPoly p 5 on cytokine production by intraperitoneal macrophages stimulated with rPoly p 5: (**A**) IL-6, (**B**) IL-10, (**C**) IFN-γ. The bars represent the mean ± (S.E.M) of cytokine concentrations detected in the cell cultures supernatants of BALB/c mice intraperitoneal macrophages stimulated with the proinflammatory stimulus (LPS at 1 μg/mL—light gray and black bars), or that received no stimulus (white and dark gray bars). Concomitantly with the addition of this stimulus, the cells were incubated with rPoly p 5 at a concentration of 1 μg/mL for 48 h (dark gray and black bars), and the controls received only cell culture medium (white and light grey bars). (*N* = 44). Please see the cytokines that showed no significant changes in Figure S1 of Supplementary Material.

The effect of rPoly p 5 in modulating the cytokine profile produced by intraperitoneal macrophages was assessed by immunoassays based on the simultaneous detection of multianalytes by flow cytometry to the cytokine’s quantification in the cell culture supernatants. Figure 3 and Figure 4 represent the data obtained from the cultures of LPS-activated macrophages (bars on the right) or not (bars on the left) with the proinflammatory stimulus (LPS—1 μg/mL) and treated with rPoly p 5 (1 μg/mL). 

In Figure 3, we can observe a reduction in the TNF-α (Panel A) and MCP-1 (Panel B) cytokines production in activated macrophages treated with rPoly p 5 compared with their respective control. In contrast, we observed an increase in IL-1β cytokine secretion when the culture was treated only with rPoly p 5, without the addition of LPS. Figure 4, panels A and B, show an increase in the production of IL-6 and IL-10 cytokines, though not significant, was submitted to 1 μg/mL of rPoly p 5, without the addition of LPS. However, the results found did not show differences compared to unstimulated controls. On the other hand, IL-1α, IL-12p70, IL-17A, IL-23, IL-27, IFN-β, and GM-CSF cytokines showed no significant changes when cultures were stimulated with rPoly p 5 compared to their respective control group, as shown in Figure S1 of Supplementary Material.

### 2.4. Co-Stimulatory Molecules and MHCII Expression

**Figure 5:**
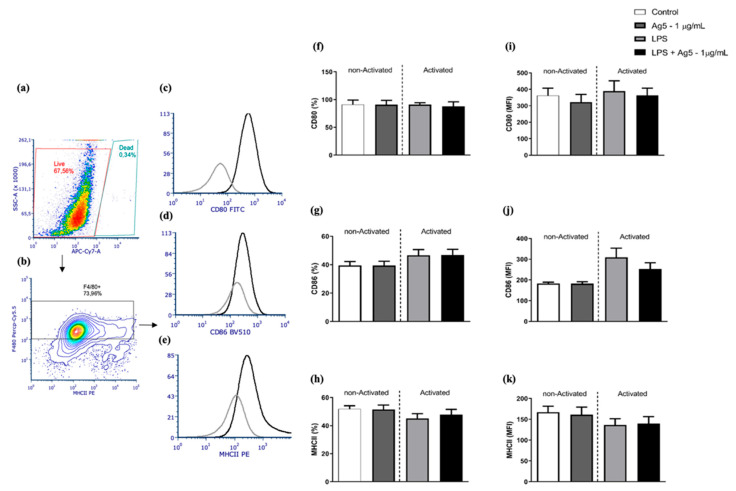
(**a**–**k**). Expression and frequency of positive cells expressing the co-stimulatory molecules CD80, CD86, and MHCII by intraperitoneal macrophages stimulated with rPoly p 5. The graphs (**a**–**e**) represent the flow cytometry assay gate strategy used. The panel (**a**), showing SSC-A vs APC-Cy7 (viability probe), was used to determine the populations of viable cells (red gate) and dead cells (blue gate). The intraperitoneal macrophages (F4/80^+^ cells—black gate) were identified in the contour plot of panel (**b**) by plotting PercP-Cy5.5 channel (F4/80) and PE channel (MHCII). The black lines in the histograms of the panels (**c**–**e**) represent the counts vs. the fluorescence intensity of the markers CD80, CD86, and MHCII, respectively. The grey lines are the measurement of their respective isotype controls. Panels (**f**–**h**) represent the mean ± (S.E.M) of the frequency of positive cells to CD80, CD86, and MHCII markers, respectively. Panels (**i**–**k**) represent the mean ± (S.E.M) of the median of the fluorescence intensity of the fluorochromes conjugated to the monoclonal antibodies triggered to CD80, CD86, and MHCII, respectively. The light grey and black bars represent cell cultures of BALB/c mice intraperitoneal macrophages which were stimulated with the proinflammatory stimulus (LPS at 1 μg/mL) and the white and dark grey bars represents cell cultures that received no stimulus. Concomitantly with the addition of this stimulus, the cells were incubated with rPoly p 5 at a concentration of 1 μg/mL for 48 h (dark grey and black bars), and the controls received only cell culture medium (white and light grey bars). The analyzes were performed in viable F4/80+ cells, as represented in the gate strategy. (*N* = 17).

To evaluate the rPoly p 5 effects on the expression of co-stimulatory and MHCII molecules, non-activated and activated (LPS stimulus) macrophages were incubated with rPoly p 5 by 48 h and then used in flow cytometry assays. In Figure 5, panels F, G, and H show the percentages of positive cells, and panels I, J, and K represent the geometric mean of fluorescence intensity to the markers CD80, CD86, and MHCII, respectively. 

We observed an increase in the fluorescence intensity of CD86 molecules in LPS-activated macrophages compared to the non-activated group (without LPS) (*p* = 0.0087). However, this difference was not seen in the frequency of cells positive for the CD86 marker. We can also observe that there were no changes in the frequencies of positive cells, or the expression of CD80, CD86, and MHCII markers in cultures treated with rPoly p 5, stimulated or not with LPS, in comparison with their respective controls.

In summary, these results indicated that the rPoly p 5 does not interfere directly in the expression of MHCII and the co-stimulatory molecules CD80 and CD86.

### 2.5. Expression of p65 Phosphorylated Form of NF-kB

**Figure 6:**
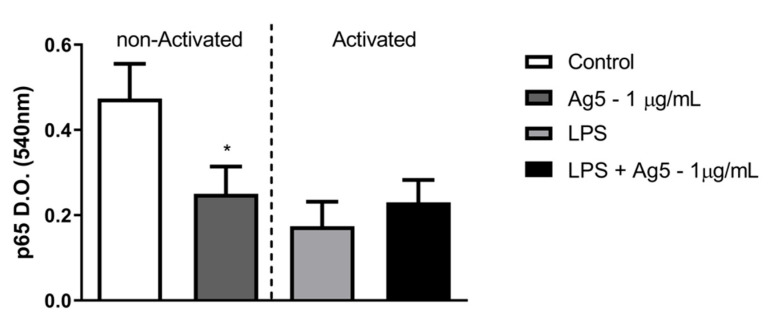
Analysis of the p 65 phosphorylated form of NF-kB in intraperitoneal macrophages. Cell cultures of BALB/c mice intraperitoneal macrophages were stimulated with the proinflammatory stimulus (LPS at 1 μg/mL—light gray and black bars) or that received no stimulus (white and dark gray bars). Concomitantly with the addition of this stimulus, the cells were incubated with rPoly p 5 at a concentration of 1 μg/mL for 48 h (dark gray and black bars), and the controls received only cell culture medium (white and light gray bars). Bars represent the mean ± S.E.M. of the optical densities (D.O.) at 540 nm wavelength. (*N* = 14) (*) *p* < 0.05—Mann–Whitney U test.

The expression of the phosphorylated form of the p65 subunit of NF-kB was evaluated by ELISA assays in LPS-activated or non-activated intraperitoneal macrophages in the absence/presence of rPoly p 5. The results in Figure 6 show that there was a decrease in the production of the phosphorylated form of the p 65 subunit of NF-kB in the macrophage nucleus, when non-activated macrophages cultures were treated with the recombinant allergen rPoly p 5. However, no significant change was observed concerning the LPS-activated group, when treated with 1 μg/mL of rPoly p 5.

## 3. Discussion

Antigen 5 is one of the most used allergens, together with phospholipase, in the specific diagnosis of allergic reactions to Hymenoptera venoms when it comes to recombinant allergens [7]. However, unfortunately, its function is still unknown. The production of recombinant allergens is an efficient strategy for obtaining large amounts of pure material relatively quickly, allowing studies on the biological potential of the allergenic protein. 

Previous works, published by our group, showed that the recombinant form of Poly p 5, obtained in the soluble form through expression in *P. pastoris*, is allergenic [1]. Based on these results, we decided to investigate whether rPoly p 5 acts as an innate immune response modulating protein in macrophages extracted from the murine peritoneum, influencing the production of cytokines, nitric oxide, and co-stimulatory and transcription factors. 

The presence of the double band of rPoly p 5 in SDS-PAGE (Figure 1) was described by King et al. [13], Vinzón et al. [12], and Schiener et al. [6], who visualized the same phenomenon for this allergen after all the purification processes (in *Vespula vulgaris*, in *Polybia scutellaris*, and also in *Solenopsis invicta*, respectively). These authors explained the observed data as a result from different extensions of post-translational processing, at the N-terminal ends of proteins, such as glycosylation, which did not prevent its use for cell assays, such as in the basophil activation test [6]. Like PLA1, the native Ag5 from *P. paulista* has N-glycosylation sites. However, there is no evidence of carbohydrate epitopes that determine cross-reactivity (CCDs) [14].

Before performing the in vitro functional tests in mouse peritoneal macrophages, screening test of the optimal dose of rPoly p 5 was conducted to verify the toxicity of this molecule in J774 and RAW 264.7 tumor cells (Figure S2—Supplementary Material). These tumor strains were used because they represent two different sources of murine macrophages lineages with a distinct inflammatory response profile [15] and provide a large numbers of cells, representing an exciting alternative to avoid unnecessary animal sacrifice. Cell viability assays with these cell lineages showed that the dosage of 2 μg/mL of rPoly p 5 has toxic activity for cells; therefore, the dose stipulated for the assays with mouse peritoneal macrophages was 1 μg/mL of rPoly p 5. These results suggest that the dosage of rPoly p 5 used in all analysis with macrophage cells in our study did not affect cell viability, ensuring that the reduction in the analyzed parameters was not due to a decrease in the number of viable cells, as discussed below.

In the present study, we showed an increase in the production of nitric oxide in macrophages cultures when these cells were stimulated with LPS at 1 μg/mL for 48 h (Figure 2). The peak of NO expression occurs 12 h after induction stimuli and declines after 72 h [16]. However, when we analyzed the stimulation with the rPoly p 5 purified protein, we found that this protein neither increases (compared to the negative control) nor inhibits (compared to the positive control) the production of NO in intraperitoneal macrophage supernatants. Previous reports described that the crude venom of the *Apis mellifera* has anti-inflammatory effects on macrophages of the RAW 267.4 and BV-2 microglia cells lineages, as it acts by inhibiting of the NF-kB pathway, thus interfering with the production of nitric oxide [17,18]. The same effect was observed with crude venom from *Bracon hebetor* and *Vespa tropica* in RAW 267.4 and BV-2 cell lines, respectively [19,20]. Our findings suggest that the allergen rPoly p 5 from *P. paulista* is not a key component in the modulation of NO production in intraperitoneal macrophage cells. Considering that venom from Hymenoptera is a complex combination of substances, as mentioned above, the behavior observed with crude venom could be the result of other venom components or even a synergic effect of different compounds.

The interaction with the venom induces the secretion of a variety of proinflammatory cytokines by macrophages. However, a detailed description of the induction of these cytokines by Hymenoptera venoms is still not clear, especially for isolated components, such as Antigen 5, whose function is still unknown. The cell culture supernatants treated with rPoly p 5 showed that the recombinant allergen decreases the expression of the proinflammatory cytokines TNF-α (*p* = 0.0297) and MCP-1 (*p* = 0.040) compared to non-treated activated macrophages. TNF-α, produced by activated macrophages, has a multifunctional biological action on the activities of target cells. This cytokine has a vasogenic property related to the infiltration of lymphocytes, neutrophils and monocytes. It also acts in the recruitment of cells to inflammation sites, thereby regulating the release of chemokines. MCP-1/CCL2 is a cytokine that has chemotactic capacity for monocytes [21]. It coordinates monocytes migration in the occurrence of physiological immunity as well as in pathological conditions, such as autoimmune disorders, which include rheumatoid arthritis, inflammatory diseases, infectious diseases, obesity, diabetes, and various types of cancer. It appears to affect leucocyte activity, including adhesion, polarization, effector molecules secretion, autophagy, death, and survival, according to new data [22].

Our data also demonstrated that stimulation with rPoly p 5 significantly increases (*p* = 0.0409) the production of the inflammatory cytokine, IL1-β, in macrophage cultures. This showed a slight in vitro increase, though not statistically relevant, in IL-6 and IL-10 cytokines compared to the non-activated control group (without LPS). The IL-1β cytokines, such as TNF-α, is a pyrogen component that is generated and released during the first phases of the immune response. It is produced by nucleated cell types, mainly monocytes, macrophages, and dendritic cells, and is one of the most significant markers of inflammatory response induction [23]. The IL-1β data, although significant in the control group as shown in Figure 3C, need further analysis, especially with a higher sensitivity IL-1β kit, as the experiment generated a lower concentration (control) than the minimum detectable concentration (2.8 pg/mL) imposed by the method used in these experiments. We also emphasize that further analysis needs to be performed with rPoly p 5 to confirm its real effect on mouse intraperitoneal macrophages in relation to the release of IL-1β. IL-6 influences the differentiation of B lymphocytes and immunoglobulin secretion [24]. Macrophages can secrete IL-6 against stimuli, such as IL-1 β and TNF-α, playing an important role in innate and adaptive immunity, with pro and an anti-inflammatory property depending on the microenvironment context [25]. IL-10 exerts anti-inflammatory and regulatory functions, as it suppresses several NK cell functions and prevents the increased expression of molecules involved in antigen presentation and lymphocyte activation [26]. 

Macrophages treated with apidaecin, a protein found in *A. mellifera* venom, increased the release of cytokines and chemokines, such as IL-1β, IL-6, IL-10, IL-12p70, TNF-α, and MCP-1. However, when apidaecin was tested on macrophage cell cultures that were stimulated with LPS, there was an inhibition of IL-6 and TNF-α. Interestingly, at high concentrations, apidaecin presented inflammatory effects, and, at low concentrations, apidaecin acted as an anti-inflammatory molecule [27]. Kaushik et al. [20] conducted studies to verify the effects of *Vespa tropica* crude venom on the production of proinflammatory cytokines in LPS-treated BV-2 cells. Exposure of the BV-2 cell to LPS resulted in elevated production of TNF-α, IL-6, and MCP-1, which were significantly reduced after the exposure of the cultures to different concentrations of the crude venom, suggesting a potent anti-inflammatory property of *V. Tropica* venom. A similar effect was reported using the venom of the *Bracon hebetor* wasp in RAW 264.7 cells treated with LPS, where there was a decrease in IL-1β, IL-1, and TNF-α cytokines, suggesting an anti-inflammatory effect [19].

This work evaluated the production of CD80, CD86, and MHCII co-stimulatory molecules by peritoneal macrophages stimulated with rPoly p 5. We showed that rPoly p 5 does not change the frequency of positive cells or the expression of CD80, CD86 and MHCII markers, neither in activated (LPS stimulated) nor in non-activated macrophages. These observations corroborate to the hypothesis that antigens with allergenic effect do not stimulate, or do so in low tone, the innate immune system.

In contrast, it was observed that in vitro stimulation of macrophages with the protein apidaecin, from *Apis mellifera* venom, leads to the upregulation of the co-stimulatory molecule CD80 expression and partial reduction in MHCII and CD86 molecule expression, in LPS-activated macrophages [27]. In our study, the intraperitoneal administration of thioglycolate medium in BALB/c mice was used as a sterile chemical irritant to induce an acute local inflammatory response, accompanied by local emigration phagocytic cells, which provide the appropriated amount of these cells to the experimental analysis. Wu et al. [28] showed that thioglycolate medium promotes an increase in co-stimulatory molecules CD80, CD86, and especially MHCII in mouse peritoneal macrophages collected after 4 days with a decline after the sixth day. As seen in Figure 5, the co-stimulatory molecules and MHCII expression in peritoneal macrophages after LPS stimulus does not differ from non-stimulated (negative control). A possible explanation to this finding could be related with the fact that the co-stimulatory molecules and MHCII molecules were highly expressed on the surface of macrophages as a consequence of thioglycolate stimulation before induction with rPoly p 5 and LPS, corroborating the data found by Wu et al. [28]. 

The nuclear factor kappa B (NF-κB) pathway is a classic inflammatory pathway activated whenever cellular stress occurs. This pathway comprises a series of transcriptional components from the cytoplasm and the nucleus, responsible for inducing the expression of several genes related to the inflammatory response [29]. Among them, are the genes encoding the growth factor GM-CSF; IL-1, -2, -6, -8, as well as TNF-α, INF-α, and β cytokines; the anti-apoptotic elements, TRAF-1, TRAF-2, c-IAP1, and c-IAP2; and others [30,31]. NF-κB is also a key transcription factor that regulates the expression of the inducible form of nitric oxide synthase (iNOS) [32]. NF-κB can be activated by proinflammatory cytokines, such as TNF-α and IL-1, T and B lymphocyte-activating factors, bacterial LPS, viral proteins, growth factors, and stress-inducing factors [33,34]. Its inhibition can also be performed in several ways, such as by non-steroidal anti-inflammatory drugs, anti-inflammatory steroid hormones, and toxins [32] As demonstrated here, there was a significant decrease (*p* = 0.0463) in the expression of the phosphorylated form of NF-κB p65 subunit in cultures of non-activated macrophages treated with rPoly 5. However, no significant change was observed in activated macrophages (LPS stimulated), with or without rPoly p 5 treatment. Results obtained with the venom of the parasitic wasp *Bracon hebetor* revealed that the crude venom suppressed, in a dose-dependent manner, the phosphorylation of all components related to the NF-κB and MAPK pathways in RAW 264.7 cells [19]. The same was reported for *A. mellifera* crude venom in cells of the same lineage, where a decrease in iNOS, COX-2, and NF-κB mRNA expression was observed. The results suggest that the anti-inflammatory effects of the venom may occur due to the inhibition of these genes expression, possibly through the suppression of NF-κB [17]. 

M1 macrophages are characterized by high expression of MHC-II, CD80, and CD86, as well as increased production of TNF-α, Il-1β, IL-6, IL-12, IL-23, and MCP-1 cytokines [35]. However, the literature suggests that there is plasticity between M1 and M2 phenotypes; depending on the microenvironmental stimulus received by cell, a change in the functional phenotype may occur [35,36]. M2 macrophages do not constitute a uniform population and are often subdivided into M2a, M2b, and M2c. M2a/c macrophage profiles show alterations in some metabolic pathways, such as arginine metabolism, leading to the production of ornithine and polyamine instead of citrulline and NO production. M2 macrophages also have a low capacity to promote antigen presentation [37]. While M1 macrophages are considered microbicides and proinflammatory (post-infectious pathogenesis), M2 macrophages are immunomodulatory (M2a and M2c) and weakly microbicide [35]. The common denominator of all three subpopulations is high IL-10 production accompanied by low IL-12 production. One of its properties is the production of Arginase-1 enzyme, which depletes L-arginine, thus suppressing T lymphocyte responses and depriving iNOS of its substrate [35]. 

In the present study, the macrophages collected from mice peritoneal cavity, after thioglycolate stimulus, possibly produced an M1 macrophage profile since thioglycolate induces inflammatory response in phagocytic cells discussed above. Here, we suggest the hypothesis that, following the stimulation with the rPoly p 5 allergen, there was a change in the phenotypical profile of the activated macrophages, converting them from M1 phenotype to M2a/c. This is corroborated by a significant decrease observed in TNF-α and MCP-1. Furthermore, the absence of the induction of nitric oxide and co-stimulatory expression by rPoly P5 could also reinforce this hypothesis However, further investigations using more specific phenotypical markers of M1 and M2 populations should be carried out to determine the influence of rPoly p 5 in the modulation of the functional profile of macrophages. 

## 4. Conclusions

Our results showed that the rPoly p 5 allergen did not alter nitric oxide production and did not modify the frequency of positive cells or expression for CD80, CD86, and MHCII markers in intraperitoneal macrophage cells of BALB/c. Otherwise, rPoly p 5 acts as a modulator of the immune response, inhibiting the production of cytokines TNF-α and MCP-1, when stimulated with LPS, and inducing the in vitro production of the cytokine IL-1β in macrophages extracted from BALB/c mice. Furthermore, rPoly p 5 significantly decreased the in vitro production of the p65-activated form of the transcription factor NF-kB in macrophages when compared to the negative control group.

Therefore, the evaluation of the pro- or anti-inflammatory potential of the rPoly p 5 allergen, through the analysis of intraperitoneal macrophage responses in mice, has not yet allowed a clear understanding of the immunomodulatory potential of this protein. However, some findings discussed here may indicate an essential role of this allergen in the polarization of functional phenotypes of M2 macrophages. In this sense, further investigations, mainly in vivo analysis, should be carried out to elucidate the biological role of Ag5 from *Polybia paulista* in the modulation of the macrophage functional profile.

## 5. Materials and Methods

### 5.1. Recombinant Antigen 5 (rPoly p 5) Obtention 

The allergen rPoly p 5 was acquired, as demonstrated by Bazon et al. [1]. Briefly, the rPoly p 5 cDNA was cloned in the pPICZαA vector and expressed in *Komagataella phaffii* (*Pichia pastoris)* X-33 cells. The soluble rPoly p 5 samples were subjected to purification by application in a prepacked column HisTrap HP™ (Ni^+2^ Sepharose™ High Performance; GE Healthcare, Danderyd, Sweden), according to the manufacturer’s instructions. Afterwards, SDS-PAGE electrophoresis tests (12%) were performed to assess the purification process effectiveness. The purified protein was resuspended in phosphate-buffered saline (PBS, pH 7.4) for cell culture experiments. 

### 5.2. SDS-PAGE and Protein Quantification

SDS-polyacrylamide gel electrophoresis (SDS-PAGE) 12% was conducted, as described by Laemmli [38], with the employment of a Mini-Protean^®^ Tetra Cell System (BioRad, Hercules, CA, USA). Following protein separation by electrophoresis, the gels were stained, either with a Coomassie Brilliant Blue R-250 (CBB) or a Pierce™ Silver Staining Kit (Thermo Fisher Scientific, Waltham, MA, USA). The purified rPoly p 5 was quantified by the modified Bradford [39] method, with a standard of bovine serum albumin solution (Sigma-Aldrich^®^, St. Louis, MO, USA).

### 5.3. Macrophage Cell Culture

Female BALB/c mice between 4 to 6 weeks old were acquired from the Multidisciplinary Center for Biological Research (CEMIB) at Campinas State University (UNICAMP). The mice were kept in the Experimental Animal Facility of the Translational Immunology Laboratory (LIT) under specific pathogen-free (SPF) conditions, in ventilated racks (Alesco, SP, Brazil) with controlled light, temperature, and humidity. The procedures involving animals and their care were carried out according to the Brazilian Committee for Animal Experimentation (COBEA) guidelines and recommendations. The Institutional Animal Experimentation and Ethics Committee (CEUA-UNICAMP) evaluated and approved all the experimental protocols (CEUA/UNICAMP. Protocol #4845-1, approval date: 7 May 2018). 

Macrophages were collected from BALB/c mices’ peritoneal cavities, which previously received an intraperitoneal injection of 3 mL of 3% thioglycolate medium fluid solution (Neogen, MI, USA) 4 days prior to the assays. The cells were transferred to sterile conical polypropylene tubes and subjected to centrifugation at 200 ×*g* for 10 min at 5 °C. The supernatant was discarded, and the pellet was resuspended in a complete culture medium (RPMI 1640, Sigma), supplemented with sodium bicarbonate (2 g/L), HEPES buffer (25 mM, Sigma-Aldrich^®^), 10 UI/mL of penicillin, 10 µg/mL of streptomycin, and 5 µg/mL of Fungizone (all from Gibco, Invitrogen Corporation, Grand Island, NY, USA). Cells were counted in a hemocytometer. For cell culture, the suspension was balanced at 1 × 10^6^ cells/mL [40].

Mouse peritoneal macrophages were seeded in a volume of 200 μL/well at a density of 2 × 10^5^ cells/well, in 96-well plates (Jetbiofil, Guangzhou Jet Bio-Filtration Co., Ltd., Guangzhou, China) and at 1 × 10^6^ cells/well in 12-well plates (Jetbiofil) being both cultured for 2 h. Debris were extracted by washing with RPMI 1640 medium lacking FBS. Complete RPMI 1640 medium was added, 200 μL per well in 96-well plates and 1 mL per well in 12-well plates. Macrophages were stimulated (activated) or not (non-activated) with LPS (1 μg/mL; LPS, *Escherichia coli*, O111:B4, Sigma), and treated with rPoly p 5 at a concentration of 1 μg/mL. The respective controls of activated or non-activated macrophages received just cell culture medium. The cultures were incubated at 37 °C, for 48 h, in humidified 5% CO_2_. After incubation, aliquots of these cells were collected for evaluation of their surface markers by flow cytometry. The cell cultures were also used for assay of NO detection, cytokines quantification, and NF-kB analysis, as described below.

### 5.4. Determination of Nitrite Concentration in Culture Supernatants 

Quantitative colorimetric tests based on the Griess reaction were used to measure the amount of nitric oxide (NO) released in cell culture supernatants [41]. Fifty microliter’s aliquots of the cell culture supernatants were incubated with 50 μL of Griess reagent (1% of sulfanilamide, 0.1% of N-(1-naphthyl ethylenediamine), and 2.5% of ortho-phosphoric acid dihydrochloride). Succeeding incubation for 10 min at room temperature, optical density was assessed, at 540 nm, in a microplate reader (Spectramax 190, Molecular Devices, Sunnyvale, CA, USA). Nitrite concentrations were determined by comparison with a calibration curve obtained with standard solutions of sodium nitrite.

### 5.5. Evaluation of CD80, CD86 and MHCII Cell Markers 

To assess the activation profile of intraperitoneal macrophages, the expression of co-stimulatory molecules CD80 and CD86 in the population of F480+ and MHCII+ cells was analyzed. For this purpose, initially, the Zombie NIR™ Fixable Viability Kit (BioLegend, San Diego, CA, USA) was used to stain the viable single-cell suspensions. Following the washing utilizing flow cytometry cell staining buffer (FCSB—phosphate-buffered saline pH 7.4 with 5% SFB, 2 mM of EDTA, and 2 mM of NaN3), the cells were labelled with anti-mouse F480 Percp-Cy5.5 (Biolegend) and anti-mouse MHCII+ APC (Biolegend) for population labelling of macrophages, anti-mouse CD80 PE antibodies (Biolegend), and anti-mouse CD86 Alexa-488 (Biolegend), following the manufacturers’ recommendations. The reading of the samples was made using FACsVerse™ Flow Cytometer (BD-Bioscience San Jose, CA, USA). The lower limit of events for the acquisitions was 10,000, gated on viable cells for each tube, ensuring that all analyses were performed only on viable cells. Non-marked cells and single-stained cells were analyzed, with the support of the antibodies previously mentioned to regulate the photomultiplier tubes (PMTs) voltage and compensation adjustments. FACSuite acquisition software version 1.0.6 (Becton Dickinson) was used to manually establish the compensation matrix. The determination of positive populations was established based on the fluorescence obtained in the samples labeled with the respective isotype control antibodies for the analyzed markers. The results were analyzed with the FCS Express V6 software (De Novo Software, Glendale, CA, USA). Figure 4 presents the gating strategy used in the analysis. The expression of CD80, CD86, and MHCII markers was evaluated using the geometric mean of the fluorescence intensity of the respective conjugated fluorochrome.

### 5.6. Quantification of Cytokines in Culture Supernatants 

Cell culture supernatants were used through multiplex quantitation system by flow cytometry for the quantification of the following cytokines: IL-1α, IL-1β, IL-6, IL-10, IL-12p70, IL-17A, IL-23, IL-27, CCL2 (MCP-1), IFN-β, IFN-γ, TNF-α, and GM-CSF. To this, the LEGENDplex^TM^ Mouse Inflammation Panel Kit (13-plex) (Biolegend, San Diego, CA, USA) was used, following the manufacturer’s instructions. The samples were analyzed in a flow cytometer, flow FACSVerse^TM^ (BD-Bioscience), and the analysis was performed with LEGENDplex^TM^ Data Analysis Software V.8 (Biolegend, San Diego, CA, USA). 

### 5.7. Evaluation of p65 Phosphorylated Subunit Form of NF-κB

The p65 phosphorylated subunit of NF-κB cells was assessed in the cultures of LPS-activate or non-activated macrophages treated with rPoly p 5, at a concentration of 1 μg/mL. Briefly, intraperitoneal macrophage cultures were seeded at 1 × 10^6^ cells/mL in 96-well plates and incubated at 37 °C containing 5% CO_2_ atmosphere and 90% humidity for 48 h. After incubation, the culture supernatants were harvested, and NFκB (p 65) labelling was performed in cell lysates obtained from the adherent cells according to the Instant One ELISA^TM^ 96-well Kit instructions (Invitrogen, Thermo Fisher Scientific). Optical density was measured on a microplate reader (SpectraMax, Molecular Devices) at 540 nm.

### 5.8. Statistical Analysis

For graphics compositions and statistical analysis, we used the GraphPad Prism software version 6.0 (La Jolla, CA, USA). Results were showed as mean ± standard error mean (S.E.M), and the Mann–Whitney U test was used to compare groups, considering a significance of *p* < 0.05.

## Supplementary Materials

The following are available online at https://www.mdpi.com/article/10.3390/toxins13120850/s1, Figure S1: The effect of rPoly p 5 on cytokine production by intraperitoneal macrophages stimulated with rPoly p 5; Figure S2: Cell viability analysis of cultured macrophage lineages J744 and RAW after treatment with different concentrations of rPoly p 5.
