# Supplementary material for: Biological and Inflammatory Effects of Antigen 5 from Polybia paulista (Hymenoptera, Vespidae) Venom in Mouse Intraperitoneal Macrophages
Source: Toxins (Basel). 2021 Nov 29;13(12):850. doi: 10.3390/toxins13120850 (PMC8703750; doi:10.3390/toxins13120850)
Supplement: Supplementary file 1 [file toxins-13-00850-s001.zip › toxins-1415081-supplementary.pdf]

Supplementary Materials: Biological and Inflammatory Effects of Antigen 5 from *Polybia Paulista* (Hymenoptera, Vespidae) Venom in Mouse Intraperitoneal Macrophages

Murilo Luiz Bazon, Luis Gustavo Romani Fernandes, Isabela Oliveira Sandrini Assugeni, Lucas Machado Pinto, Patrícia Ucelli Simioni, Ricardo de Lima Zollner and Márcia Regina Brochetto Braga

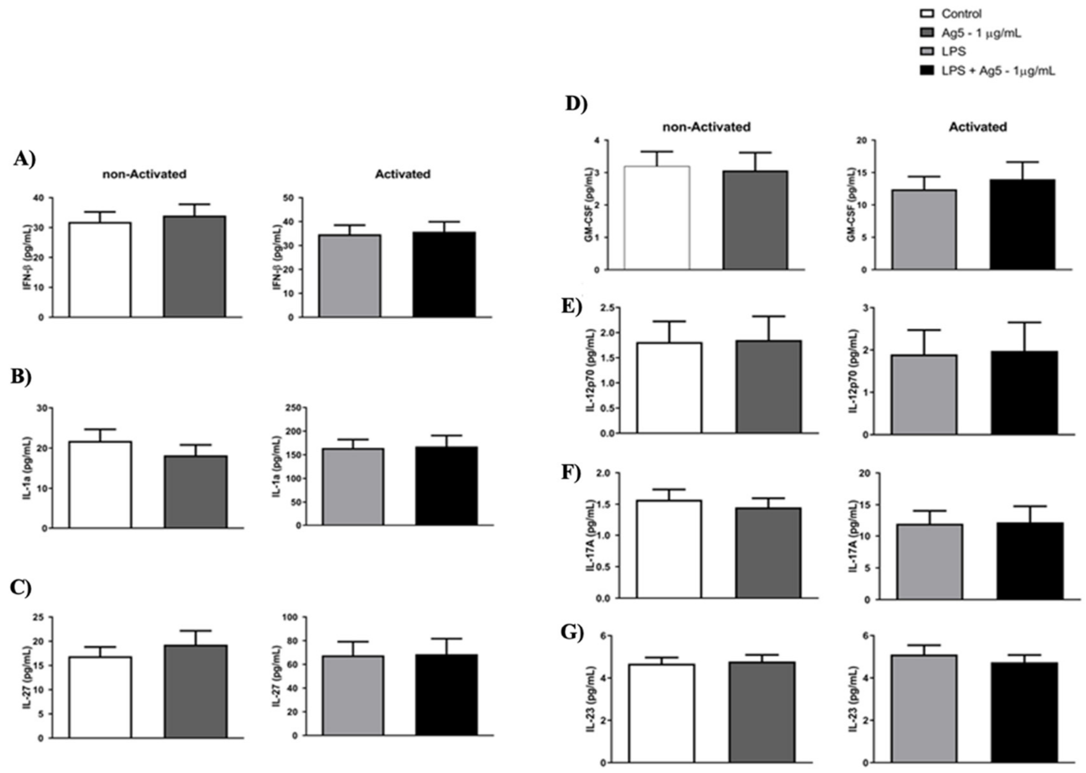

Figure S1. The effect of rPoly p 5 on cytokine production by intraperitoneal macrophages stimulated with rPoly p 5: **(A)** IFN-β, **(B)** IL1-a, **(C)** IL-27, **(D)** GM-CSF, **(E)** IL-12p70, **(F)** IL-17A, **(G)** IL-23. The bars represent the mean ± (S.E.M) of cytokine concentrations detected in the cell cultures supernatants of BALB/c mice intraperitoneal macrophages stimulated with the proinflammatory stimulus (LPS at 1 µg/mL—light gray and black bars) or that received no stimulus (white and dark gray bars). Concomitantly with the addition of this stimulus, the cells were incubated with rPoly p 5 at a concentration of 1 µg/mL for 48 h (dark gray and black bars), and the controls received only cell culture medium (white and light grey bars). (*N* =4 4) (*) $p < 0.05$—Mann-Whitney U test.

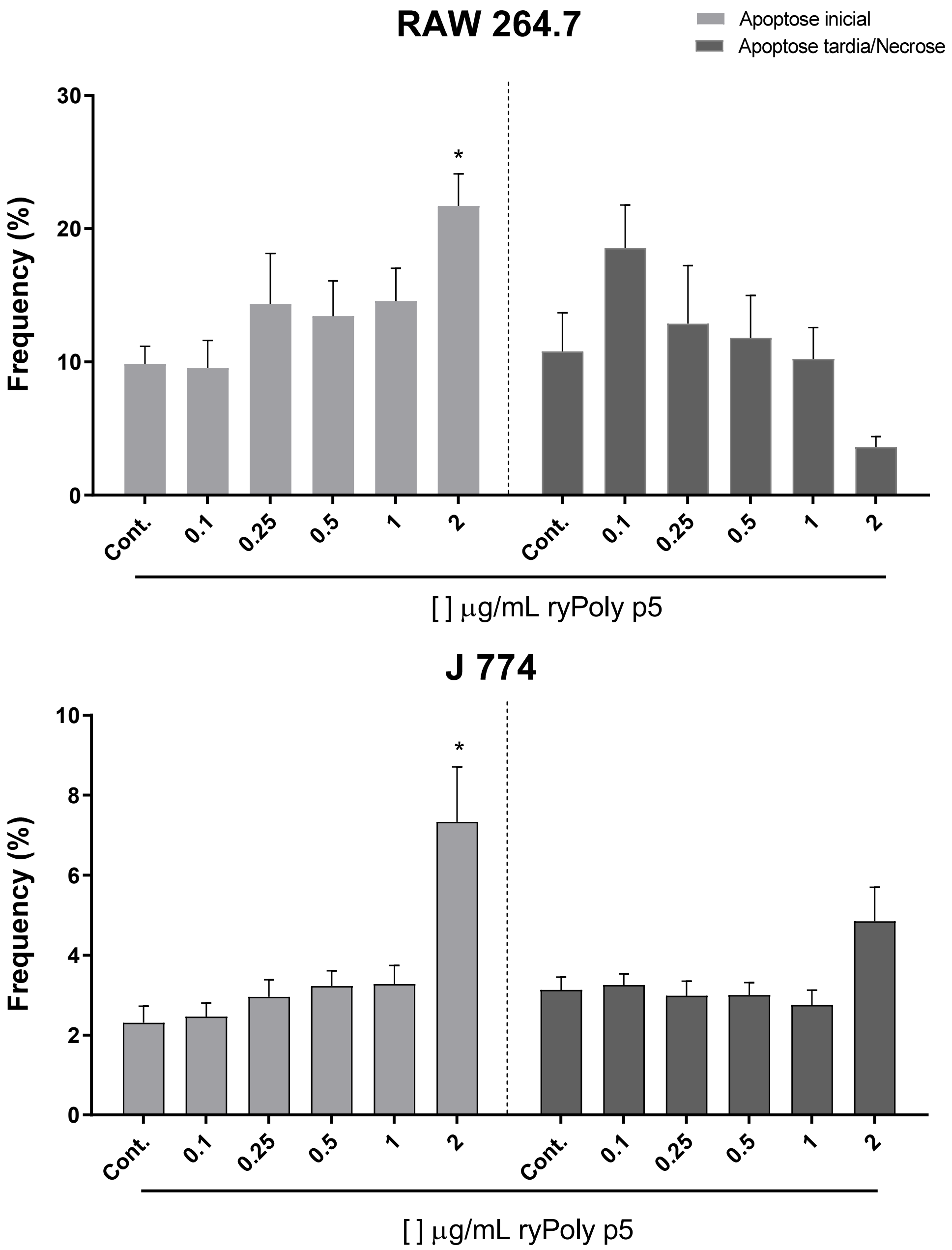

Figure S2: Cell viability analysis of cultured macrophage lineages J744 and RAW after treatment with different concentrations of rPoly p 5 (0.1 µg/mL; 0.25 µg/mL; 0.5 µg/mL; 1 µg/mL; 2 µg/mL). The light grey bars indicate the percentage of viable cells in the initial phase of apoptosis and dark grey bars represents the late phase of apoptosis (necrosis). Bars represent mean ± standard deviation.

Material and Methods

Cell Viability Assessment

The assessment of viability and cell death mechanisms were performed by labeling the cells using the Fitc Annexin V apoptosis detection Kit, with 7-Aminoactinomycin D (7AAD) (Biolegend, San Diego, CA, USA), according to the manufacturer's recommendations. The samples were acquired in a flow cytometer, flow FACSVerse™ (BD-Bioscience) located at the Center for Comprehensive Care for Women's Health CAISM -UNICAMP and the analysis were performed with the FCS Express V6 software (De Novo Software, Glendale, CA, USA). Results were expressed as the percentage of viable cells (double negative), in early apoptotic phase (AnnexinV+/7AAD-) and in late apoptosis/necrosis (AnnexinV+/7AAD+).
